# Interaction of Heat Shock Protein Cpn10 with the Cyclin E/Cdk2 Substrate Nuclear Protein Ataxia-Telangiectasia (NPAT) Is Involved in Regulating Histone Transcription*

**DOI:** 10.1074/jbc.M115.659201

**Published:** 2015-10-01

**Authors:** Li Ling Zheng, Fei Ya Wang, Xiao Xia Cong, Yue Shen, Xi Sheng Rao, Dao Sheng Huang, Wei Fan, Peng Yi, Xin Bao Wang, Lei Zheng, Yi Ting Zhou, Yan Luo

**Affiliations:** From the ‡ Department of Biochemistry and Molecular Biology,; § Key Laboratory of Tissue Engineering and Regenerative Medicine of Zhejiang Province, Center for Stem Cell and Regenerative Medicine, and; ¶ Department of Orthopaedic Surgery, Second Affiliated Hospital, Zhejiang University School of Medicine, Hangzhou, Zhejiang 310058, China,; the ‖ Department of Abdominal Tumor Surgery, Zhejiang Cancer Hospital, Hangzhou, Zhejiang 310022, China, and; the ** Department of Surgery and Sidney Kimmel Cancer Center, Johns Hopkins University School of Medicine, Baltimore, Maryland 21231

**Keywords:** cell cycle, cell proliferation, cyclin, heat shock protein (HSP), histone, NPAT, Cpn10, cell cycle, histone transcription

## Abstract

Precise modulation of histone gene transcription is critical for cell cycle progression. As a direct substrate of Cyclin E/CDK2, nuclear protein ataxia-telangiectasia (NPAT) is a crucial factor in regulating histone transcription and cell cycle progression. Here we identified that Cpn10/HSPE, a 10-kDa heat shock protein, is a novel interacting partner of NPAT. A pool of Cpn10 is colocalized with NPAT foci during G_1_ and S phases in nuclei. Gain- and loss-of-function experiments unraveled an essential role of Cpn10 in histone transcription. A conserved DLFD motif within Cpn10 was critical for targeting NPAT and modulating histone transcription. More importantly, knockdown of Cpn10 disrupted the focus formation of both NPAT and FADD-like interleukin-1β-converting enzyme-associated huge protein without affecting Coilin-positive Cajal bodies. Finally, Cpn10 is important for S phase progression and cell proliferation. Taken together, our finding revealed a novel role of Cpn10 in the spatial regulation of NPAT signaling and disclosed a previously unappreciated link between the heat shock protein and histone transcription regulation.

## Teaser

**Background:** NPAT is critical for histone transcription and cell cycle progression.

**Results:** A novel NPAT-interacting protein, Cpn10/HSPE, is critical for histone transcription and focus formation of NPAT.

**Conclusion:** Heat shock protein Cpn10 is a novel regulator for histone transcription and cell proliferation.

**Significance:** Our findings reveal a previously unappreciated role of heat shock protein in regulating histone transcription.

## Introduction

In eukaryotic cells, to avoid intertwisting, the newly synthesized DNA needs to rapidly wrap around the histone core to assemble chromatin during S phase in the cell cycle. Therefore, DNA synthesis and histone gene transcription need to be precisely coupled at the G_1_/S phase cell cycle transition (1). The S phase-specific histones responsible for the DNA package are termed replication-dependent histones and include the core histones H2A, H2B, H3, and H4 and the linker histone H1 (2). It is well documented that histone mRNA levels increase severalfold when cells enter S phase, providing enough histone scaffolds for packing nascent DNA (3). Disruption of histone gene expression will disturb DNA synthesis and result in cell cycle arrest (4–6). Therefore, the characterization of cellular factors involved in modulating histone gene transcription represents a crucial step toward a better understanding of the mechanism underlying cell cycle regulation.

As a direct downstream substrate of cyclin E/CDK2, nuclear protein ataxia-telangiectasia locus (NPAT/p220)^4^ is one of the most crucial factors in regulating histone gene transcription (7, 8). Phosphorylation of NPAT at the G_1_/S boundary is required for activating histone gene expression (9). Down-regulation of NPAT suppresses histone gene expression and affects cell cycle progression (3). Because no DNA-binding motif was identified in the NPAT sequence, it is proposed that other factors are required for coordinating the functions of NPAT in regulating histone transcription and the cell cycle. A series of NPAT-interacting proteins were subsequently identified. For example, FADD-like interleukin-1β-converting enzyme-associated huge protein (FLASH) exhibits complete co-localization with NPAT, and the interaction of NPAT and FLASH is required for S phase progression (10). The histone nuclear factor P (HiNF-P) is another NPAT-interacting partner that enables the association of NPAT and the histone gene promoter. When the NPAT-HiNF-P interaction was disrupted, both histone H4 gene activation and cell cycle progression were compromised (11). In addition, the transient interaction between NPAT and CREB-binding protein (CBP)/p300 acetyltransferase at the G_1_/S boundary promotes S phase entry (12). All of these findings suggest that the identification of novel NPAT-interacting proteins is crucial for understanding the spatiotemporal regulation of the cell cycle and histone synthesis machinery.

The majority of heat shock proteins are reported to function as a chaperone or chaperonin in the cytoplasm. Here we discovered, by yeast two-hybrid screening, that Chaperonin 10 (Cpn10), also designated heat shock protein 10 (Hsp10), is a novel binding partner of NPAT. It is well documented that Cpn10 forms a complex with Cpn60 (Hsp60) in all bacteria and eukaryotic cell organelles, including mitochondria and chloroplasts (13, 14). This chaperonin complex is critical for assisting the folding of newly synthesized proteins. Studies in mammalian cells have revealed that Cpn10 is a multifunctional protein. Cpn10 protects myocytes against simulated ischemia and reoxygenation-induced cell death by inhibiting Ras signaling (15). It not only regulates bone marrow differentiation but also modulates the apoptosis pathway in cardiac muscle cells by regulating the expression of the Bcl-2 and Bax proteins (15–17). A very recent study has shown that down-regulation of Cpn10 increased mitochondrial fragmentation and potentiated mitochondrial dysfunction in neuroblastoma cells (18). In addition, Cpn10 has been found to be up-regulated in various tumors, but the physiological relevance is unclear (18–20). Interestingly, we found that a pool of Cpn10 colocalized with NPAT in nuclei, although previous studies have shown that Cpn10 is a chaperonin localized to mitochondria (21). A conserved DLFD motif was identified in Cpn10 for targeting NPAT. Importantly, our results showed that Cpn10 is critical for focus formation of NPAT, histone transcription, and cell proliferation. Taken together, we demonstrated that the Cpn10-NPAT interaction is critical for the regulation of histone transcription, highlighting a unique role of heat shock proteins in regulating the cell cycle and cell proliferation.

## Experimental Procedures

### 

#### 

##### Cell Culture and Transfection

HeLa cells were cultured in DMEM supplemented with 10% FBS, 2 mm l-glutamine, 100 units/ml penicillin, and 100 mg/ml streptomycin (all from Hyclone Laboratories, Logan, UT). Cells in 6-well plates were transfected with TransIT-LT1 (Mirus Bio) or Lipofectamine 2000 (Invitrogen) according to the protocol of the manufacturer.

##### Yeast Two-hybrid Assay

A yeast two-hybrid assay was performed with the N-terminal fragment of NPAT (1–821 amino acids) as the bait against a human cDNA library. We followed the detailed protocol from the Matchmaker yeast two-hybrid system user manual from Clontech.

##### Antibody

The antibodies used were as follows: anti-Cpn10 (catalog no. sc-20958, Santa Cruz Biotechnology, SPA-110, Stressgen), anti-NPAT (catalog nos. sc-136007 and sc-32359, Santa Cruz Biotechnology), anti-FLASH (catalog no. sc-9088; Santa Cruz Biotechnology), anti-Coilin (catalog no. ab11822, Abcam), anti-FLAG epitope (Sigma-Aldrich), anti-HA epitope (Invitrogen), and anti-Tubulin (HuaAn Biotechnology).

##### RNA Interference

Cells at 30–40% confluency were transfected with 100 nm siRNA using Lipofectamine RNAiMAX (Invitrogen) according to the protocol of the manufacturer. Cells were harvested for analysis 24 h (for qPCR) or 48 h (for immunofluorescence or Western blot analysis) after transfection. Sequences of siRNAs were as follows: siCpn10#1, AGGAAAGGGUGGAGAGAUUdTdTo; siCpn10#2, GGACAAGCGUUUAGAAAGUdTdT; and siNPAT, GGGUUUGCGAAGUGAGAAAdTdT. The sequences of control siRNA was UUCUCCGAACGUGUCACGUdTdT.

##### Construction of Expression Plasmids

Full-length cDNA of Cpn10 was cloned into a HA-, GFP-, and FLAG-tagged expression vector, pXJ40 (Dr. E. Manser, Institute of Molecular and Cell Biology, Singapore). The Cpn10 mutant form was generated by PCR mutagenesis techniques with the following primer, in which three amino acids, LFD, were replaced by triple alanines: TTAGAAAGTTTCTTCCAGCAGCAGCACGAGTATTGGTTGAAAGGA and CCCAAGCTTTCAGTCTACGTACTTTCC. The sequences of the second round of PCR primers were CCCGATATCATGGCAGGACAAGCGTTTAGAAAGTTTCTTCC and CCCGGATCCATGGCAGGACAAGCGTTTAGAAAGTTTCTTCC. All plasmids were purified using the Axygen miniprep kit for use in transfection experiments. *Escherichia coli* strain DH5α was used as a host for propagation of the clones.

##### Quantitative RT-PCR

Total RNA was isolated with the RNeasy kit (Qiagen). Reverse transcription was carried out with the SuperScript III reverse transcriptase kit (Invitrogen). qPCR reactions using the KAPA SYBR FAST qPCR MasterMix kit (Kapabiosystems) were performed in triplicate with the following primers: histone H2B forward primer, CAGTGCTATGCCAGAGCCAGCGAA; histone H2B reverse primer, CTGTTTACTTAGCGCTGGTGTACTTGGTGA; histone H4 forward primer, GCGGCGGCGTCAAGCGTATT; and histone H4 reverse primer, AAGGGCCGTTGGTTTTGCGG. Actin was used to normalize gene expression. Its specific primers were as follows: Actin forward primer, GCCGACAGGATGCAGAAGGAGATC; Actin reverse primer, AAGCATTTGCGGTGGACGATGGA.

##### Immunoprecipitation Studies and Western Blot Analyses

Control cells or cells transfected with expression plasmids were lysed in lysis buffer (150 mm sodium chloride, 50 mm Tris (pH 7.3), 0.25 mm EDTA, 1% (w/v) sodium deoxycholate, 1% (v/v) Triton X-100, 0.2% sodium fluoride, 0.1% sodium orthovanadate, and a mixture of protease inhibitors from Roche Applied Science). Lysates were immunoprecipitated with anti-FLAG M2 beads (Sigma), and the associated proteins were separated on SDS-PAGE and probed with anti-HA (for cotransfection experiments). Samples were run in SDS/PAGE gels and analyzed by Western blotting with anti-HA or anti-FLAG.

##### GST Pulldown Assay

Both the wild-type and mutant form of Cpn10 were constructed in a pGEX vector with a GST tag. Both constructs, together with the control vector expressing GST alone, were transformed into BL21 bacteria. Highly expressed GST fusion proteins were dialyzed with lysis buffer (0.2 mm EDTA, 2 m NaCl, 1% Triton X-100, 1.5 mm PMSF, and 1.5 mm DTT) and purified on glutathione-Sepharose beads (GE Healthcare). Subsequently, these GST beads, with respective GST fusion proteins, were incubated with whole-cell lysate of NPAT-overexpressing HeLa cells at 4 °C for 2–3 h and finally subjected to Western blot analysis.

##### Immunofluorescence and Direct Fluorescence Studies

Cells were seeded on coverslips in a 24-well plate, transfected with various expression constructs for 24–36 h, and then stained for immunofluorescence detection using confocal fluorescence microscopy or visualized directly for cells expressing GFP-tagged proteins, as described previously (22). FLAG-tagged proteins were detected with monoclonal anti-FLAG followed by Texas red or FITC dye-conjugated goat anti-mouse IgG (Invitrogen). Filamentous actin was detected by rhodamine-phalloidin (Molecular Probes). The images were collected using a Zeiss 510 Meta laser-scanning microscope equipped with a ×60 lens. The detector gain was first optimized by sampling various regions of the coverslip and then fixed for each specified channel. When set, the detector gain value was kept constant throughout the image acquisition process.

##### Cell Proliferation Assay

The proliferation of HeLa cells was determined using a standard 3-(4,5-dimethylthiazol-2-yl)-5-(3-carboxymethoxyphenyl)2-(4-sulfophenyl)-2*H*-tetrazolium (MTS)viability test (CellTiter AQueous One Solution cell proliferation assay kit, Promega, Madison, WI) according to the instructions of the manufacturer. All samples were read in quadruplicate.

##### Colony Formation Assay

3 × 10^3^ HeLa cells were seeded in triplicate in 6-cm dishes and maintained in DMEM supplemented with 10% fetal bovine serum. The growth medium was changed every 3 days. After 14 days, the resulting colonies were rinsed with PBS, fixed with methanol for 10 min, and stained with crystal violet. Colony formation efficiency was evaluated by counting individual foci for three independent experiments.

##### Luciferase Assay

HeLa cells were transfected with an H4 promoter-luciferase (firefly) reporter together with a Cpn10 HA plasmid or empty vector. A *Renilla* luciferase construct under the control of the SV40 promoter was used as an internal reference in this experiment. One day after transfection, HeLa cells were lysed, and the luciferase activities were analyzed to assess H4 transcription *in vivo*. The H4-promoter-luciferase reporter construct was provided by Dr. Jiyong Zhao (24).

## Results

### 

#### 

##### Cpn10 Interacts with NPAT

**FIGURE 1.**
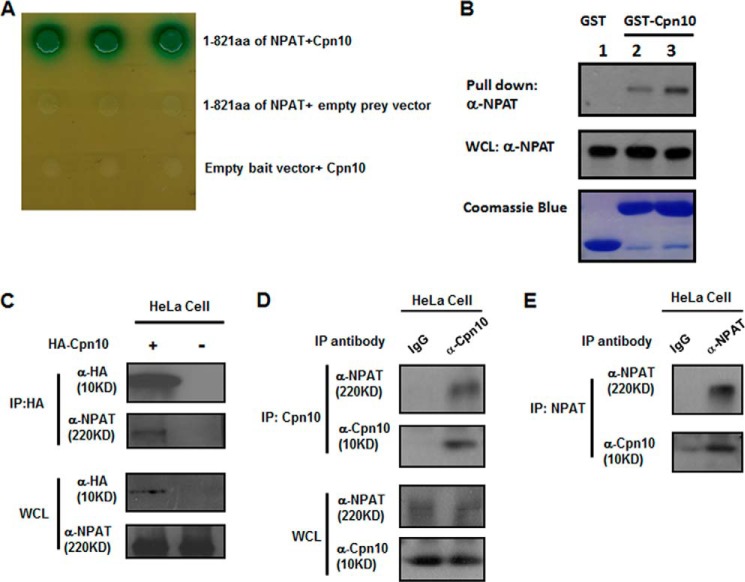
**Cpn10 is a novel binding partner of NPAT.** *A*, Cpn10 interacted with NPAT in a yeast two-hybrid assay. The N-terminal fragment of NPAT (1–821 amino acids) was cloned into the yeast plasmid pGBKT7. Yeast colonies containing both Cpn10 and the N terminus of NPAT plasmids could grow in high-stringency medium and turned blue in the presence of 10 μg/ml X-α-gal. No growth was observed in yeast transformed with only the Cpn10 plasmid plus empty pGBKT7 or pGBKT7-NPAT_1–821_ plus empty pGADT7. *aa*, amino acids. *B*, 20 μl of control GST protein (*lane 1*) or 20 μl (*lane 2*) or 40 μl (*lane 3*) GST-Cpn10 fusion protein was incubated with NPAT-overexpressing HeLa cell lysate for a pulldown assay and then Western-blotted with NPAT-specific antibody (*top panel*). The amounts of GST and GST-Cpn10 used in this experiment are indicated by Coomassie Blue staining (*bottom panel*). *WCL*, whole-cell lysate. *C*, HeLa cells transfected with the HA-Cpn10 plasmid or a control empty plasmid. The whole-cell lysates were immunoprecipitated (*IP*) with anti-HA beads and then Western-blotted with NPAT-specific antibody. *D*, whole-cell lysates of HeLa cells were immunoprecipitated with Cpn10-specific antibody or an IgG control and then Western-blotted with either NPAT or Cpn10 antibodies. *E*, whole-cell lysates of HeLa cells were immunoprecipitated with NPAT-specific antibody or IgG control and then Western-blotted with either Cpn10 or NPAT antibodies.

It has been reported previously that the N terminus of NPAT is required for histone gene activation (11, 23). To identify novel interacting partners of NPAT in regulating histone gene transcription, the N terminus of NPAT (1–821 amino acids) was used as a bait to conduct a yeast two-hybrid assay. A positive clone corresponding to Cpn10/HSP10 was identified (Fig. 1*A*). To confirm the interaction between Cpn10 and NPAT, we carried out a pulldown experiment by using the recombinant GST-Cpn10 fusion protein. NPAT could be pulled down by the GST-Cpn10 fusion protein, and an increased amount of Cpn10 can pull down more NPAT (Fig. 1*B*). This indicates that Cpn10 interacts with NPAT *in vitro*. Next we examined the Cpn10-NPAT interaction in mammalian cells by performing a co-immunoprecipitation assay. HA-tagged Cpn10 was transfected into HeLa cells, and empty vector was used as a negative control. Endogenous NPAT protein was found to be immunoprecipitated by the HA-Cpn10, but no interaction was detected in the control lane (Fig. 1*C*). Furthermore, cell lysates from HeLa cells were immunoprecipitated with Cpn10 antibodies and probed for endogenous NPAT. Western blot analysis showed the presence of both endogenous Cpn10 and NPAT in the precipitate (Fig. 1*D*). Consistently, the reciprocal immunoprecipitates obtained with NPAT antibody contained Cpn10 (Fig. 1*E*). All of these data suggest that Cpn10 is a novel binding partner of NPAT.

##### Colocalization of Cpn10 and NPAT during G_1_ and S Phases in Nuclei

**FIGURE 2.**
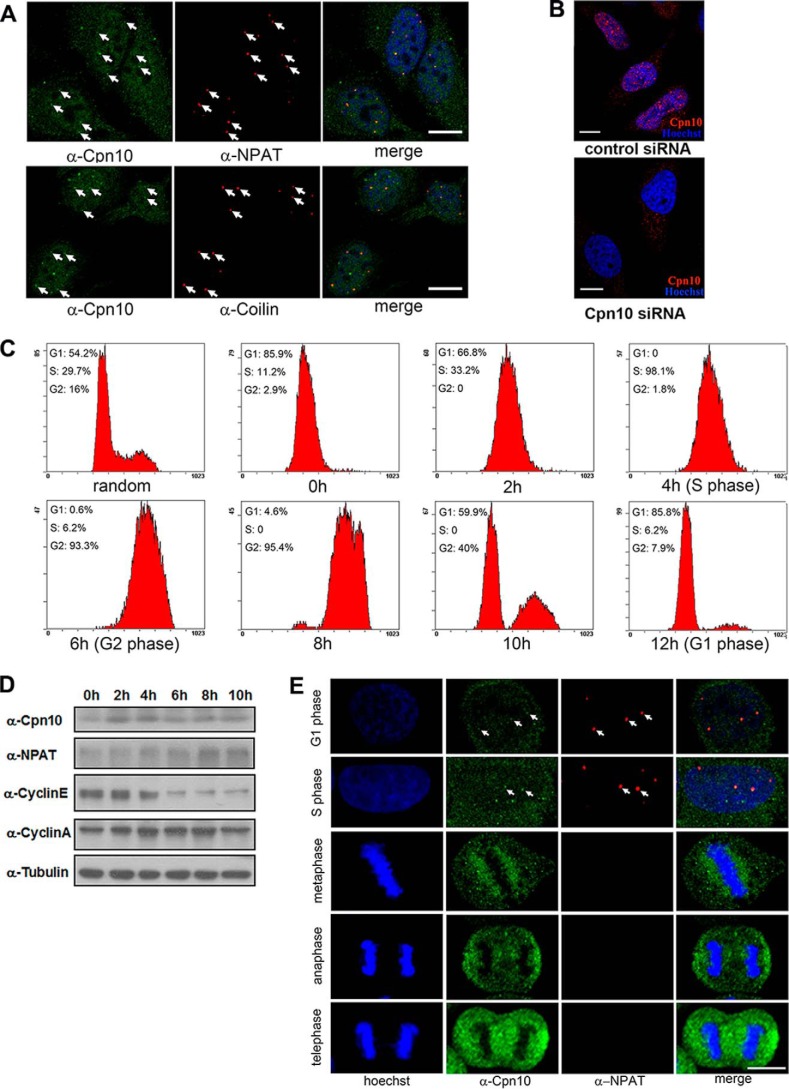
**Nuclear co-localization of Cpn10 and NPAT.** *A*, HeLa cells were fixed and co-immunostained either with anti-Cpn10 (*green*) and anti-NPAT (*red*) antibodies (*top panel*) or with anti-Cpn10 (*green*) and anti-Coilin (*red*) antibodies (*bottom panel*). Cell nuclei were visualized by Hoechst staining. *Scale bars* = 10 μm. *B*, HeLa cells transfected with control siRNA or Cpn10 siRNA for 48 h, followed by staining with Cpn10 antibody for immunofluorescence analysis. Nuclei were visualized by Hoechst staining. *Scale bars* = 10 μm. *C*, HeLa cells were synchronized at the G_1_/S boundary and then harvested 0, 2, 4, 6, 8, and 10 h after release. FACS was carried out to show cell cycle distribution profiles of synchronized HeLa cells at the indicated time point. *D*, HeLa cells were synchronized at the G_1_/S boundary and then harvested 0, 2, 4, 6, 8, and 10 h after release. Cell lysates were subjected to Western blot analysis with the indicated antibodies. *E*, HeLa cells in different phases of the cell cycle were fixed and double-stained for endogenous Cpn10 (*green*) and NPAT (*red*), followed by confocal fluorescence microscopy analysis. G_1_, S, or mitosis cells were obtained 0, 4, or 8–10 h after release, respectively. Nuclei were visualized by Hoechst staining. *Scale bar* = 10 μm.

It is well documented that functional NPAT forms foci in the nuclei of cells. Some of the NPAT foci colocalize with Cajal bodies, whereas most NPAT colocalized with FLASH in histone locus bodies (23, 24). This is consistent with our observations (data not shown). Cpn10 has been reported previously to be a mitochondrial localization chaperone (21). The interaction between Cpn10 and NPAT raised the possibility that a pool of Cpn10 might colocalize with NPAT in nuclei. We tested this hypothesis by detecting the endogenous Cpn10 together with the staining of NPAT. As shown in Fig. 2*A*, endogenous Cpn10 is present in both the cytoplasm and nucleus. The nuclear pool of Cpn10 was enriched in small nuclear foci, which were disrupted in Cpn10 knockdown cells, demonstrating the specificity of the Cpn10 antibody used (Fig. 2*B*). Most of Cpn10 foci colocalized with those of NPAT (Fig. 2*A*, *top panel*), and part of the endogenous Cpn10 is coincident with coilin, the marker of Cajal bodies (Fig. 2*A*, *bottom panel*).

Because the focus formation of NPAT exhibited a cell-cycle dependent manner (24), it was worth checking whether Cpn10 showed the same manner of nuclear localization as NPAT. To obtain synchronized HeLa cells, a double thymidine block was used to arrest cells at the G_1_/S boundary. Successful cell cycle synchronization was judged by FACS profiles (Fig. 2*C*). Western blot analysis showed that the expression of Cpn10 in S phase cells was higher than that in mitotic cells (Fig. 2*D*). Endogenous Cpn10 in the nuclei exhibited foci with NPAT both in G_1_ phase (0-h time point) and S phase (4 h after release) but not in mitosis, during which the NPAT signal disappeared from nuclei (Fig. 2*E*). Our observations showed that Cpn10 exhibited co-localization with NPAT during G_1_ and S phases in nuclei.

##### Cpn10 Is Required for Histone Gene Transcription

**FIGURE 3.**
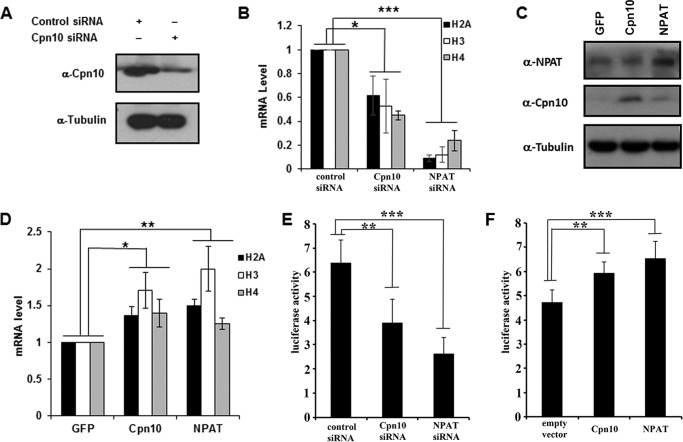
**Activation of histone gene transcription by Cpn10.** *A*, Western blotting was performed to check the efficiency of Cpn10 siRNA by using Cpn10 antibodies after Cpn10 siRNA or control siRNA transfection. Equal amounts of protein loading were confirmed by tubulin antibody. *B*, quantification of histone H2A, H3, and H4 expression in Cpn10 and NPAT down-regulated HeLa cells using real-time quantitative PCR. *C*, GFP, Cpn10, or NPAT plasmids were overexpressed in HeLa cells for analyzing the expression of histone H2A, H3, and H4 as shown in *D*. Cell lysates were subjected to Western blot analysis with the indicated antibodies. *D*, quantification of histone H2A, H3, and H4 expression in Cpn10- and NPAT-overexpressing HeLa cells. The GFP plasmid was used as a negative control. *E*, luciferase reporter assays for the H4 promoter in HeLa cells treated with siRNAs of the control, NPAT, or Cpn10 for 48 h. *F*, luciferase reporter assays of the H4 promoter in HeLa cells transfected with wild-type Cpn10, NPAT, or HA empty vector. *, *p* < 0.05; **, *p* < 0.01; ***, *p* < 0.001.

NPAT plays critical roles in regulating histone gene transcription (24). Given that Cpn10 interacted with NPAT, we postulated that Cpn10 is also involved in regulating histone transcription. To test this hypothesis, we used Cpn10-targeted siRNA to knock down Cpn10 expression and checked the transcription of histone H2B, H3, or H4 by real-time quantitative PCR. The siRNA sequence targeting Cpn10 substantially reduced the endogenous Cpn10 protein level in HeLa cells (Fig. 3*A*). In the real-time PCR assay, the levels of histone transcripts in cells treated with either Cpn10 siRNA or NPAT siRNA were decreased significantly (Fig. 3*B*). We next examined the effects of overexpression of Cpn10 on histone gene transcription. Overexpression of Cpn10 or NPAT results in a modest increase in both proteins (Fig. 3*C*). Compared with the negative control, both Cpn10- and NPAT-overexpressing cells displayed enhanced histone transcription levels (Fig. 3*D*).

We further confirmed the roles of Cpn10 in modulating histone transcription activity by luciferase assay. To this end, we either knocked down Cpn10 or overexpressed Cpn10 plasmid in HeLa cells hosting a plasmid containing the H4 promoter driving luciferase expression. The luciferase assay showed that the transcription of histone H4 was down-regulated in cells treated with Cpn10 siRNA and up-regulated in Cpn10 overexpressing cells (Fig. 3, *E* and *F*). Furthermore, knockdown of NPAT reduced histone expression more than knockdown of cpn10. This is consistent with our qRT-PCR analysis (Fig. 3*B*). Therefore, we concluded that Cpn10 is a novel regulating protein for histone gene transcription.

##### The DLFD Motif within Cpn10 Is Critical for Binding NPAT and Modulating Histone Transcription

**FIGURE 4.**
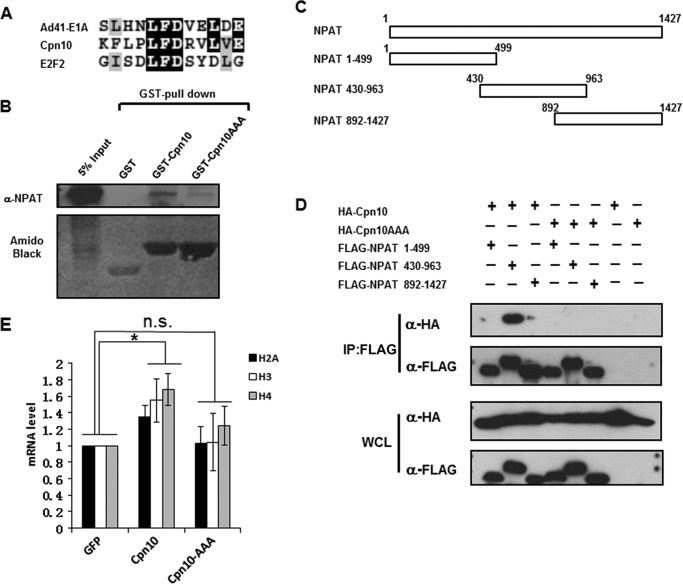
**Identification of the DLFD motif within Cpn10.** *A*, alignment of sequences revealed a conserve DLFD motif in Cpn10, Ad41-E1A, and E2F2. Identical amino acid residues are shaded *black*, and similar residues are shaded *gray. B*, the GST-Cpn10 fusion protein, AAA mutant, and control GST protein was incubated with NPAT-overexpressing HeLa cell lysate and then Western-blotted with NPAT-specific antibody (*top panel*). Equal amounts of GST, GST-Cpn10, and the GST-Cpn10 AAA mutant were used in this experiment, as indicated by the Amido Black-stained SDS-PAGE gel (*bottom panel*). *C*, full-length NPAT was dissected into three fragments as indicated. *D*, three distinct fragments of NPAT were tagged with FLAG and co-transfected with either HA-Cpn10 or the HA-Cpn10 AAA mutant in HeLa cells. Lysates were immunoprecipitated (*IP*) with anti-FLAG M2 beads and then Western-blotted with FLAG or HA antibodies. *E*, quantification of histone H2A, H3, or H4 expression in wild-type Cpn10- and AAA mutant-overexpressing HeLa cells. GFP was used as a negative control. *: *p* < 0.05; *n.s.*, not significant.

We next set out to identify the NPAT binding site in Cpn10. Previous studies revealed a four-residue DLFD motif as a conserved NPAT binding sequence that is present in several NPAT-interacting proteins (25). Among the four amino acids of the DLFD motif, the LFD sequence is much conserved, whereas the first amino acids of the motifs of different NPAT-interacting proteins can often vary. We therefore performed sequence alignment and found that N terminus of Cpn10 contains a putative DLFD motif in which the first amino acid is proline rather than aspartic acid (Fig. 4*A*). To further explore the role of this motif of Cpn10 in binding NPAT, we changed the conserved amino acids LFD to alanines (AAA mutant) and performed a pulldown assay. Fig. 4*B* shows that GST-Cpn10 can bind NPAT, whereas the interaction between the GST-AAA mutant and NPAT was much reduced. Therefore, we determined that the DLFD motif within Cpn10 is critical for interaction with NPAT.

We further asked which region of NPAT is required for binding Cpn10. To this end, three NPAT truncation mutants, as shown in Fig. 4*C*, were prepared and tested for their ability to co-immunoprecipitate HA-tagged Cpn10 or the Cpn10 AAA mutant. As shown in Fig. 4*D*, region 430–963 interacts with the Cpn10 wild type, whereas two other mutants failed to do so. Consistent with Fig. 4*B*, none of the three truncation mutants showed interaction with the Cpn10 AAA mutant (Fig. 4*D*). Taken together, our data show that the DLFD motif within Cpn10 is critical for binding the 430–963 region of NPAT.

Because both Cpn10 and NPAT played critical roles in modulating histone transcription, it is of interest to check whether Cpn10 regulates histone transcription via interaction with NPAT. Therefore, the histone transcription levels were tested in cells transfected with Cpn10 wild-type or the AAA mutant plasmid because the DLFD motif is critical for Cpn10 to interact with NPAT. As shown in Fig. 4*E*, the Cpn10 AAA mutant lost the ability to enhance histone transcription.

##### The Nuclear Focus Formation of NPAT Is Cpn10-dependent

**FIGURE 5.**
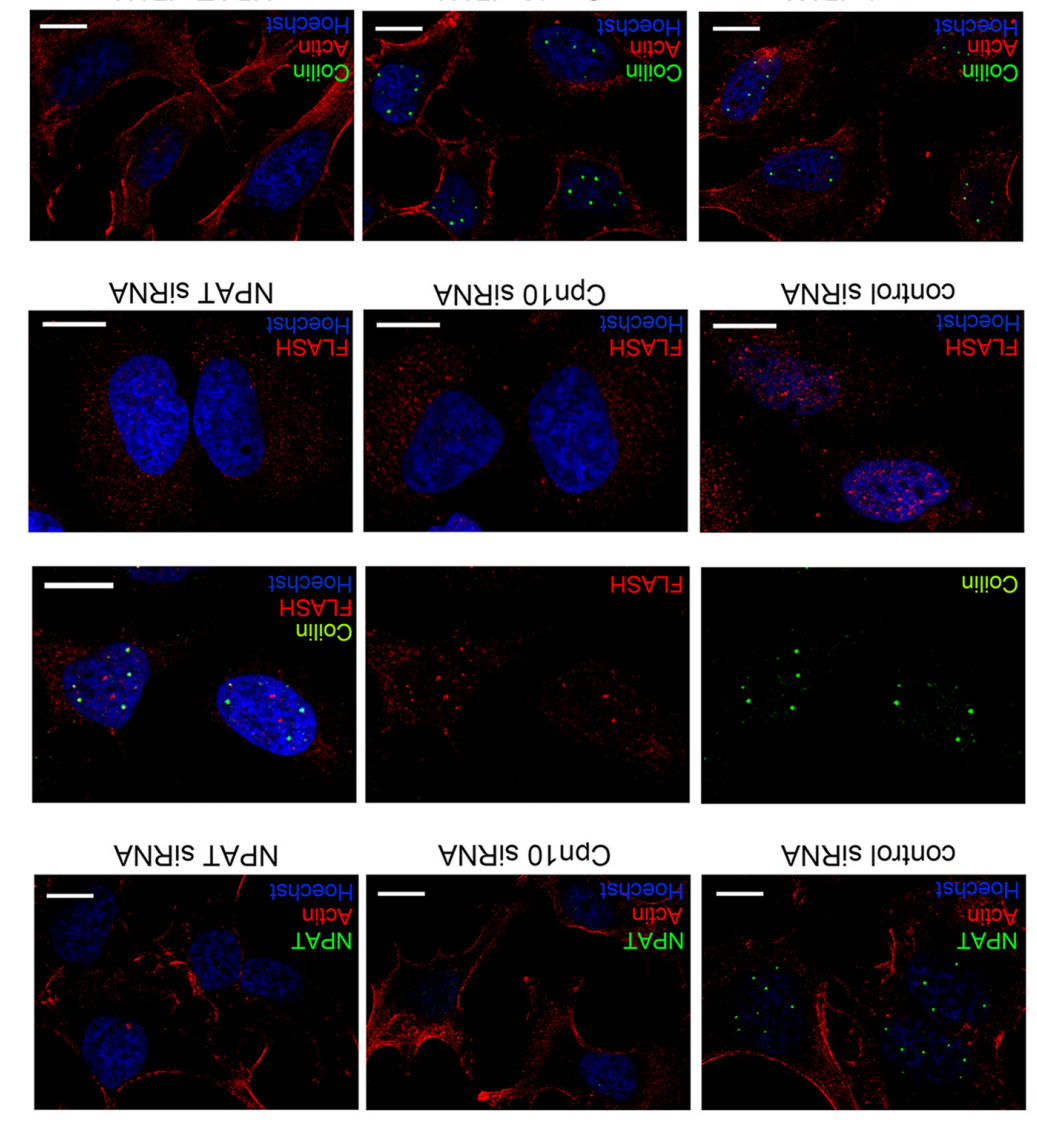
**Localization of NPAT at the Cajal body relies on Cpn10.** *A*, HeLa cells were fixed and immunostained with NPAT antibody (*green*) after 48-h transfection with the indicated siRNA. The actin filaments were detected by direct staining with rhodamine-conjugated phalloidin (*red*). *B*, HeLa cells were fixed and immunostained with FLASH (*red*) and Coilin (*green*) antibodies. Nuclei were visualized by Hoechst. *C*, HeLa cells were fixed and immunostained with FLASH antibody (*red*) after 48-h transfection with the indicated siRNA. Nuclei were visualized by Hoechst. *D*, HeLa cells were fixed and immunostained with Coilin antibody (*green*) after 48-h transfection with the indicated siRNA. The actin filaments were detected by direct staining with rhodamine-conjugated phalloidin (*red*). Nuclei were visualized by Hoechst. *Scale bars* in *A–D* = 10 μm.

The focus formation of NPAT in nuclei is critical for its function (24, 26). We therefore hypothesized that Cpn10 might be important for maintaining the cellular localization of NPAT. To test this possibility, we stained the endogenous NPAT in Cpn10 siRNA-treated HeLa cells. Random siRNA- and NPAT siRNA-treated cells were used as negative or positive controls, respectively. In the nuclei of cells transfected with random siRNA, endogenous NPAT is concentrated in a few foci that were not observed in either NPAT- and Cpn10 siRNA-treated cells (Fig. 5*A*). This demonstrates that Cpn10 is critical for the focus formation of NPAT.

NPAT and FLASH are components of the histone locus body, whereas Coilin is a widely used marker for Cajal bodies (27, 28). Knockdown of NPAT disrupts FLASH focus formation and vice versa (27, 28). Similar to NPAT, endogenous FLASH displayed limited colocalization with Coilin (Fig. 5*B*). Because Cpn10 is required for NPAT focus formation, we proposed that Cpn10 reduction could disrupt the FLASH-positive nuclear body. To test this hypothesis, we stained endogenous FLASH in siRNA-treated HeLa cells. As shown in Fig. 5*C*, no FLASH-positive nuclear body was detected after knockdown of Cpn10. However, coilin foci were intact in Cpn10 siRNA-treated cells (Fig. 5*D*). These result demonstrate that Cpn10 is not required for Coilin-decorated Cajal body formation but critical for the focus formation of both NPAT and FLASH.

##### Cpn10 Regulates Cell Cycle and Cell Proliferation

**FIGURE 6.**
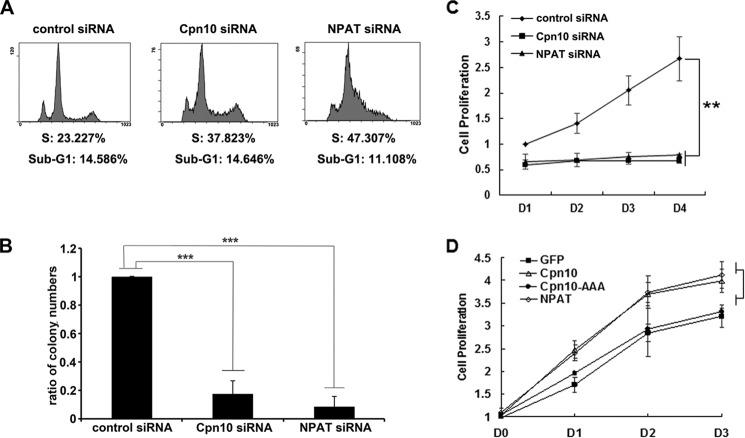
**Regulation of Cpn10 in the cell cycle and cell proliferation.** *A*, FACS data for the percentage of cells at different cell phases in HeLa cells transfected with the indicated siRNA. *B*, quantification of the colony formation assay of HeLa cells transfected with the indicated siRNA. The *y* axis shows the ratio of colony numbers of Cpn10 siRNA- or NPAT siRNA-treated HeLa cells to control siRNA-treated cells. ***, *p* < 0.001. *C*, MTS viability assay of HeLa cells transfected with the indicated siRNA. **, *p* < 0.01. *D*, MTS viability assay of HeLa cells with overexpression of the indicated proteins. GFP was used as a negative control.

NPAT plays critical roles in cell cycle regulation, and down-regulation of NPAT results in S phase arrest (3). We therefore tested whether depletion of Cpn10 also affects cell cycle progression in HeLa cells. A propidium iodide FACS assay exhibited S phase arrest in both Cpn10 and NPAT down-regulated cells (Fig. 6*A*). It has been reported previously that Cpn10 is up-regulated during carcinogenesis (19, 20), but the physiological significance is elusive. This cell cycle defect in Cpn10-silenced cells indicates a potential role of Cpn10 in regulating cell proliferation. We first determined this possibility by cell colony formation assay to examine relative cell growth rates. We discovered that colony formation was reduced significantly in cells with down-regulation of either Cpn10 or NPAT (Fig. 6*B*). Next we checked the roles of Cpn10 in regulating cell proliferation by MTS assay. Consistent with our colony formation assay, we found that Cpn10 down-regulated cells were incapable of reducing MTS to formazan, which has an absorbance maximum at 490–500 nm (Fig. 6*C*), confirming that down-regulation of Cpn10 inhibited cell proliferation. In contrast, more formazan products were detected in Cpn10-overexpressing cells (Fig. 6*D*), showing that up-regulation of Cpn10 enhanced cell proliferation. Interestingly, the Cpn10 AAA mutant lost the capability of enhancing cell proliferation, implicating that the DLFD motif is critical for regulating both histone gene transcription and cell proliferation (Fig. 6*D*). Taken together, we conclude that Cpn10 regulates the cell cycle and cell proliferation.

## Discussion

### 

#### 

##### Regulation of NPAT by Interacting Partners

NPAT is a key regulator to coordinate histone gene expression during the G_1_/S phase transition. Therefore, identifying signaling proteins regulating NPAT functions helps to understand the underlying molecular mechanism. This was initiated by the finding of the interaction between NPAT and Cyclin E/CDK2. Cyclin E/CDK2 kinase phosphorylates NPAT at the G_1_/S phase transition (9). This posttranslational modification of NPAT is required for the association of NPAT with the histone gene promoter to initiate gene transcription (7, 9, 24). Besides phosphorylation, acetylation might play roles in modulating NPAT function. This was suggested by the finding that NPAT transiently interacts with CBP/p300 histone acetyltransferase and contains a potential substrate sequence for CBP/p300 (12). Further biochemical analysis is required to confirm this hypothesis. Interestingly, no evidence shows that NPAT could directly bind to the histone gene promoter, although it played critical roles in activating histone gene transcription. This was explained by the finding that NPAT links directly to a histone nuclear factor, HiNF-P (11). Down-regulation of HiNF-P reduces the association of NPAT with the histone H4 promoter and impairs its transcription (11). Further study has revealed that exogenous expression of HiNF-P enhances the stabilization of NPAT (29). Here we identified chaperonin Cpn10 as a novel NPAT-interacting protein. Cpn10 regulates histone transcription and the cell cycle. Importantly, inhibition of Cpn10 expression results in the lost signal of NPAT foci, whereas the coilin foci remain intact. Our study, together with others, revealed that there are multilayer regulatory mechanisms controlling the functions of NPAT in histone transcription and cell cycle progression.

##### Roles of Heat Shock Proteins in Nuclei

Appropriate subcellular localization is critical for proteins to perform their functions. Different heat shock proteins have been found to localize in divergent subcellular compartments. For example, Hsp70-9/Grp75 serves as a mitochondrial chaperone to maintain appropriate mitochondria protein folding (30). HSP70/HSPA1 localizes on the lysosomal membrane to facilitate importing proteins to be degraded (31). Similarly, isoforms of HSP90 members have been identified in distinct subcellular compartments, including the endoplasmic reticulum and mitochondrial matrix (32). Interestingly, beside cytoplasm localization, accumulating evidence suggests that heat shock proteins also play a role in nuclei. For example, mSti1 (co-chaperone murine stress-inducible protein 1) contains a nuclear localization signal that allows it to translocate between nuclei and the cytoplasm (33). Furthermore, Hsp70 has been identified by us as a component of OCA-S (Oct-1 coactivator in S phase), a nuclear protein complex activating histone H2B transcription (34). However, there still remains the question of the cellular and physiological significance of the nuclear localization of heat shock proteins. Cpn10 is classically considered a mitochondrial co-chaperonin (13). A very recent study has detected the nuclear presence of Cpn10, but the physiological significance is elusive (35). Here we showed that Cpn10/HSPE interacts with NPAT to regulate histone transcription in nuclei. This finding disclosed a previously unappreciated link between the nuclear heat shock protein and cell cycle control. It is therefore of interest to further explore the roles of other heat shock proteins, especially Hsp70, in nuclei.

The Cajal body is one of the most studied nuclear bodies, and Coilin is considered its marker (27). A previous study showed partial colocalization between NPAT and Coilin, whereas it has been proposed recently that the nuclear NPAT- and FLASH-positive foci represent the histone locus body, a distinct nuclear body for regulating histone transcription, rather than a Cajal body (27, 28, 36). Here we showed that Cpn10 reduction disrupts the focus formation of both FLASH- and NPAT-positive foci but not the Colin-decorated body. This is in line with the roles of Cpn10 in regulating histone transcription because the histone locus body is involved in histone pre-mRNA processing. The interaction between the Cajal body and the histone locus body is still elusive, although it has been reported that both NPAT and FLASH are important for Cajal body formation (28, 37). Therefore, it remains to be elucidated why knockdown Cpn10 does not affect Coilin. Down-regulation of Cpn10 might activate certain unidentified protective machinery, which awaits further investigation. We wish to follow up on this interesting question in a subsequent investigation.

##### Cpn10 and Cancer

Previous studies have shown that Cpn10 is a multifunctional protein that plays roles in regulating mitochondrial structure maintenance, bone marrow differentiation, and myocyte protection. Interestingly, accumulating documents reveal the overexpression of Cpn10 in a variety of tumors, including prostate cancer, large bowel cancer, exocervical cancer, and mantle cell lymphoma (17, 19, 20, 38). Cancer cells contain a large amount of mutated or overexpressed oncoproteins that rely on the Hsp70-Hsp90 chaperone machinery to avoid misfolding and degradation (39). It has been suggested that Cpn10 plays an active role in modulating the cell signaling network in cancer rather than act as a passive component in protein folding (13). However, the exact role of Cpn10 in tumors is still elusive. Here we showed that depletion of Cpn10 caused S phase accumulation, whereas overexpression of Cpn10 enhances cell proliferation (Fig. 6), suggesting that the rapid growth of cancer cells may be related to the high level of Cpn10. Furthermore, depletion of either Cpn10 or NPAT mediates identical reduction in cell proliferation, whereas cpn10 knockdown causes a modest accumulation in S phase compared with NPAT knockdown (Fig. 6). This might be due to mitochondrion dysfunction induced by Cpn10 knockdown because Cpn10 is also important for maintaining mitochondrial homeostasis (18). These findings confirm that Cpn10 is a multifunctional protein and raises the possibility that Cpn10 could be a potential anti-cancer drug target.

## Author Contributions

L. L. Z. and Y. T. Z. designed and coordinated the study. L. L. Z., Y. T. Z., and Y. L. wrote the paper. L. L. Z., W. F. Y., and X. X. C. performed and analyzed the immunofluorescence assays. L. Z., L. L. Z., F. Y. W., X. X. C., Y. S., D. S. H., and X. S. R. performed and analyzed the protein-protein interaction experiments. P. Y., W. F., and X. B. W. designed and constructed vectors for expression. All authors analyzed the results and approved the final version of the manuscript.

## Footnotes

4 The abbreviations used are:

NPAT: nuclear protein ataxia-telangiectasia
FLASH: FADD-like interleukin-1β-converting enzyme-associated huge protein
HiNF-P: histone nuclear factor P
qPCR: quantitative PCR
MTS: 3-(4,5-dimethylthiazol-2-yl)-5-(3-carboxymethoxyphenyl)2-(4-sulfophenyl)-2*H*-tetrazolium
CREB: cAMP response element-binding protein.
